# Cultivation of Cryopreserved Human Dental Pulp Stem Cells—A New Approach to Maintaining Dental Pulp Tissue

**DOI:** 10.3390/ijms231911485

**Published:** 2022-09-29

**Authors:** Wang Wang, Ming Yan, Ghazal Aarabi, Ulrike Peters, Marcus Freytag, Martin Gosau, Ralf Smeets, Thomas Beikler

**Affiliations:** 1 Department of Periodontics, Preventive and Restorative Dentistry, University Medical Center Hamburg-Eppendorf, 20246 Hamburg, Germany; 2 Department of Oral and Maxillofacial Surgery, University Medical Center Hamburg-Eppendorf, 20246 Hamburg, Germany; 3 Department of Oral and Maxillofacial Surgery, Division of Regenerative Orofacial Medicine, University Medical Center Hamburg-Eppendorf, 20246 Hamburg, Germany

**Keywords:** human dental pulp stem cells, methodology, dimethyl sulfoxide, cryopreservation, cellular differentiation, teeth

## Abstract

Human dental pulp stem cells (hDPSCs) are multipotent mesenchymal stem cells (MSCs) that are capable of self-renewal with multilineage differentiation potential. After being cryopreserved, hDPSCs were reported to maintain a high level of proliferation and multi-differentiation abilities. In order to optimize cryopreservation techniques, decrease storage requirements and lower contamination risks, the feasibility of new whole-tooth cryopreservation and its effects on hDPSCs were tested. The survival rates, morphology, proliferation rates, cell activity, surface antigens and differentiation abilities of hDPSCs isolated from fresh teeth were compared with those of one-month cryopreserved teeth in 5% and 10% DMSO. The data of the present study indicated that the new cryopreservation approach did not reduce the capabilities or stemness of hDPSCs, with the exception that it extended the first appearance time of hDPSCs in the teeth that were cryopreserved in 10% DMSO, and reduced their recovery rate. With the novel strategy of freezing, the hDPSCs still expressed the typical surface markers of MSCs and maintained excellent proliferation capacity. Three consecutive weeks of osteogenic and adipogenic induction also showed that the expression of the key genes in hDPSCs, including lipoprotein lipase (LPL), peroxisome proliferator-activated receptor-γ (PPAR-γ), alkaline phosphatase (ALP), runt-related transcription factor 2 (RUNX2), type I collagen (COL I) and osteocalcin (OSC) was not affected, indicating that their differentiation abilities remained intact, which are crucial parameters for hDPSCs as cell-therapy candidates. These results demonstrated that the new cryopreservation method is low-cost and effective for the good preservation of hDPSCs without compromising cell performance, and can provide ideas and evidence for the future application of stem-cell therapies and the establishment of dental banks.

## 1. Introduction

In recent years, as research on stem-cell therapy and tissue engineering has intensified, the preservation and application of mesenchymal stem cells (MSCs) has become a hot research topic. MSCs are adult stem cells with self-renewal, a high proliferating ability and multi-differentiation potential, and they have a wide range of applications in the fields of stem-cell biology, vascular tissue engineering, and other areas of regenerative medicine [1]. Among them, human dental pulp stem cells (hDPSCs) are regarded as a reliable source due to their excellent self-renewal, proliferation capacity, and multipotent differentiation ability [2]. hDPSCs were first identified by Gronthos in the year 2000 [3]. They express the specific surface biomarkers of mesenchymal stem-cell antigens such as STRO-1, CD29, CD44, CD59, CD73 and CD90, while CD19, CD24, CD34 and CD45 are not expressed [4,5]. It is reported that hDPSCs can be differentiated into various cell types in vitro, including adipocytes, chondrocytes, neurons, myocytes, and the most important for bone reconstruction in maxillofacial surgeries, osteoblasts [6,7,8,9,10]. Moreover, hDPSCs have a high differentiation potential in odontogenic lineages, and compared with bone marrow cells, a stronger ability to differentiate and proliferate [11,12]. Govindasamy et al. found that hDPSCs can even be differentiated into islet-like cell aggregates [13]. Dasari et al. found that DPSCs might be an appropriate source for therapeutic uses in neurological pathologies such as spinal cord injuries [14]. The repair and rebuilding of neuronal tissue [15,16,17,18], osseous tissue [19], hepatic tissue [16], muscle tissue [20] as well as salivary glands [21] are among the possible medicinal applications depending on DPSCs. Another significant benefit of hDPSCs is their easy accessibility: dental pulp cells can be conveniently isolated from extracted teeth, which might frequently be regarded as biological trash [22]. According to statistics, pulp tissue from interrupted third molars was most frequently used as a source of DPSCs [23]. In transplantation medicine, employing autologous cells can protect the regenerated tissue of donors from being rejected by the immune system by establishing histocompatibility banks [24]. Hence, if hDPSCs can be preserved in cell banks throughout therapy when their donors are both young and healthy, then there will be a stable supply of stem cells with excellent biological activities if their donors require them for regenerative therapies in the future.

The conventional cryopreservation process of dental pulp mesenchymal cells includes tooth disinfection, pulp extraction, cell isolation, cell proliferation, and cryopreservation [25]. However, dental clinics are not usually equipped with a research laboratory that ensures the sterile handling and storage of freshly extracted teeth. Before the hDPSC isolation, it is advantageous if the entire tooth is frozen and dental pulp is kept in an aseptic environment, namely pulp cavities, so that the possibility of sample contamination is minimized. Furthermore, in most cases it is a logistical and thus time-consuming effort to transport the extracted teeth immediately following extraction to a laboratory that is able to establish primary cell cultures.

So, we propose the immediate cryopreservation of whole teeth at −80 °C following extraction. Compared with the conventional cryopreservation procedure, this strategy can notably reduce the costs of cryopreservation—because the cell isolation step is omitted before clinical application—and storage in liquid nitrogen is unnecessary. Such limited processing might be more suitable for storing samples that do not have any imminent intentions for growth or usage. In addition, the majority of the cryopreservation methods can only be carried out in laboratories. However, the hDPSCs can only remain active in vitro for up to five days after teeth extraction [26]. The extra time needed to transport the tissue can cause damage to the cells and thus reduce the quality of the hDPSCs. To overcome this difficulty, the application of this novel method may extend the feasible period of hDPSCs in the event that samples cannot be handled in time. Therefore, the present study aimed to develop and evaluate a procedure that simplifies the storage of freshly extracted teeth intended to be used for hDPSCs. Dental pulp cells can be freshly preserved when they are most active, which may be more beneficial to the future use of these cells than long-term transportation.

## 2. Results

### 2.1. Analysis of Results

#### 2.1.1. The Impact of the Novel Cryopreservation Method on Cell Multiplication and Expansion

**Figure 1:**
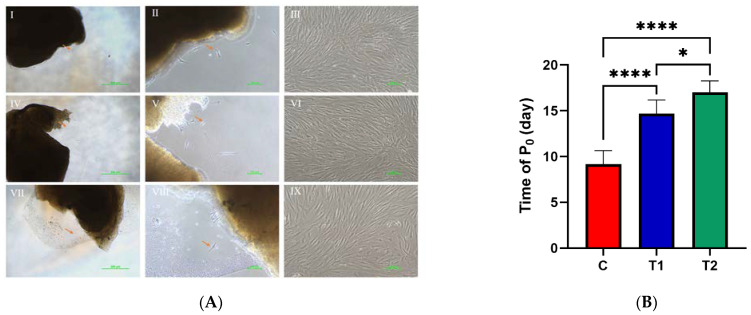
Morphology of dental pulp cells in different preservation strategy groups. (**A**) Morphology of dental pulp tissue and cells at the primary and the third generation. Ⅰ–Ⅲ: C group—Primary cells emerged from tissue blocks (×40; ×100) and the cells from the third generation after passage (×100). Ⅳ–Ⅵ: T1 group—Primary cells emerged from tissue blocks (×40; ×100) and the cells from the third generation after passage (×100). Ⅵ–Ⅸ: T2 group—Primary cells emerged from tissue blocks (×40; ×100) and the cells from the third generation after passage (×100). (**B**) The first appearance time of dental pulp cells in different groups: The first appearance time of dental pulp cells in the frozen groups was significantly longer than in the C group, and the first appearance time in the T2 frozen group was significantly longer than that in T1 group. The cryopreservation approach led to a longer period for cells to grow out of the tissue blocks. * *p* < 0.05, **** *p* < 0.0001.

Dental pulp cells were inspected under a microscope every day to observe the cell morphology. The healthy cells resembled triangular or spindle shapes, with dark cytoplasm and a distinct nucleus. After the assessment of the morphology of the DPSCs by two trained examiners, Cohen’s kappa coefficients of the T1 and T2 groups were κ = 88.9% and 83.3%, respectively, indicating almost perfect inter-observer agreement on the consistency of the cellular morphology compared with the C group (Figure 1A). The dental pulp cells in the T2 frozen group took the longest time (16.830 ± 1.472 days) until the cells were initially observed to grow and attach. A significantly longer outgrowth time of the DPSCs in T1 group (14.670 ± 1.506 days) was observed compared to the C group (9.170 ± 1.472 days) (* *p* < 0.01) (Figure 1B).

#### 2.1.2. Effects of New Cryopreservation Strategy on the Primary Cell Yield

**Figure 2:**
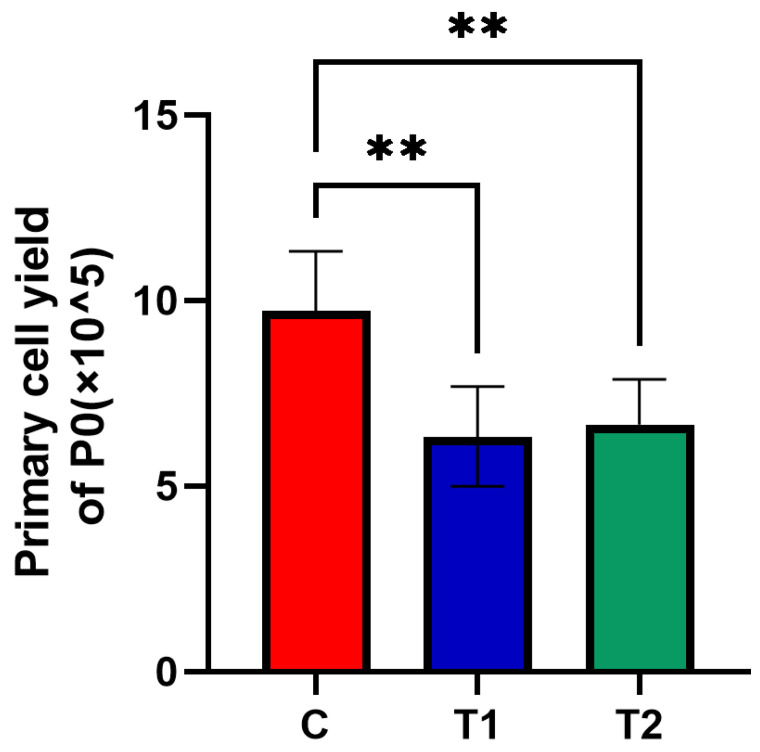
Effects of new cryopreservation strategy on the primary cell yield. The new cryopreservation strategy dramatically decreased the number of harvested primary cells compared with the C group (** *p* < 0.01).

A significantly larger number of DPSCs was collected in the C group ((9.725 ± 1.601) × 10^5^) compared with the other two groups. The number of primary dental pulp cells harvested in T1 ((6.333 ± 1.341) × 10^5^) and T2 ((6.658 ± 1.229) × 10^5^) frozen group was smaller compared with the C group (* *p* < 0.05) (Figure 2).

#### 2.1.3. Identification of Specific Stem-Cell Markers

**Figure 3:**
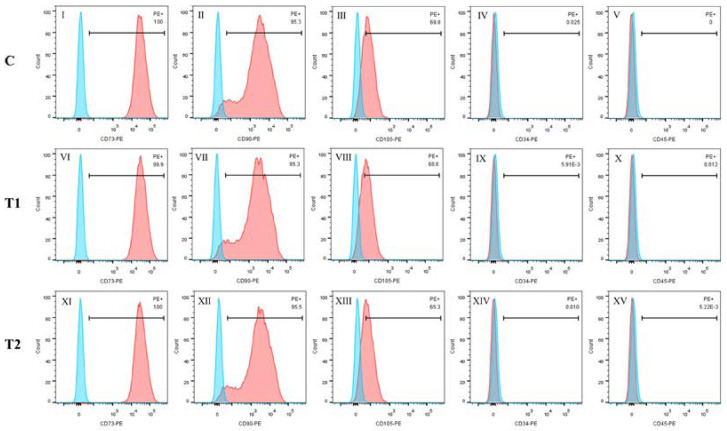
Identification of MSCs by flow cytometry in each group. Ⅰ–Ⅴ: C group; Ⅵ–Ⅹ: T1 group; Ⅺ–XV: T2 group. The expression of CD73, CD90 and CD105 was positive; otherwise, CD45 and CD34 expression was negative in the three groups.

CD34, CD45, CD73, CD90 and CD105 were detected through flow cytometry in the three groups: CD73, CD90 and CD105 were abundantly expressed, while CD34 and CD45 were not strongly expressed. (Figure 3).

#### 2.1.4. Effects of New Cryopreservation Strategy on the Cell Survival Rate after Trypan Blue and Live–Dead Staining

**Figure 4:**
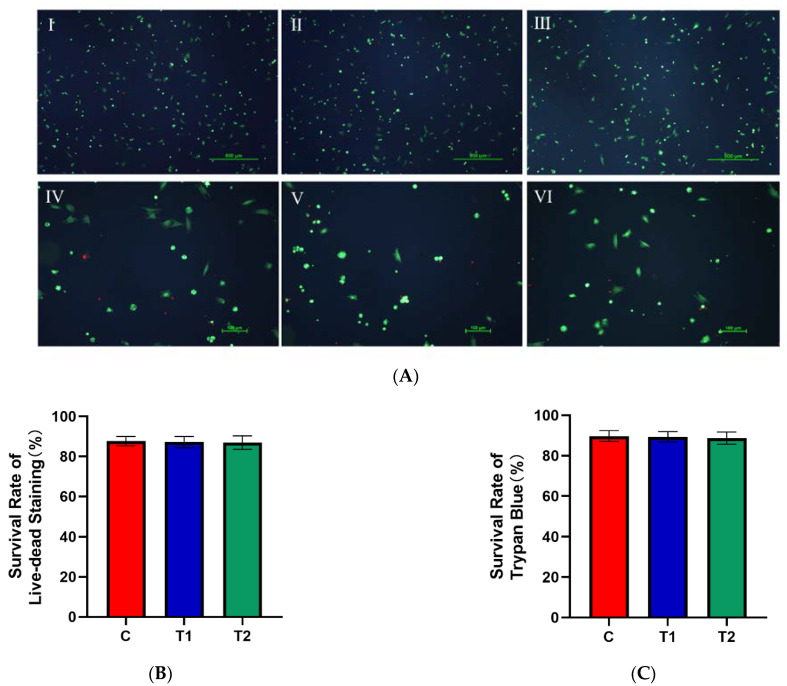
Effects of new cryopreservation strategy on the cell survival rate. (**A**) Live–dead staining results of dental pulp cells. Ⅰ: C group (×40); Ⅱ: T1 group (×40); Ⅲ: T2 group (×40); Ⅳ: C group (×100); Ⅴ: T1 group (×100); Ⅵ: T2 group (×100). (**B**) The survival rate of live–dead staining in each group. (**C**) The survival rate of Trypan Blue staining in each group.

There was no significant difference in the cell survival rate among the three groups (*p* > 0.05) (Figure 4B,C).

#### 2.1.5. Effects of New Cryopreservation Strategy on the Primary Cell Proliferation

**Figure 5:**
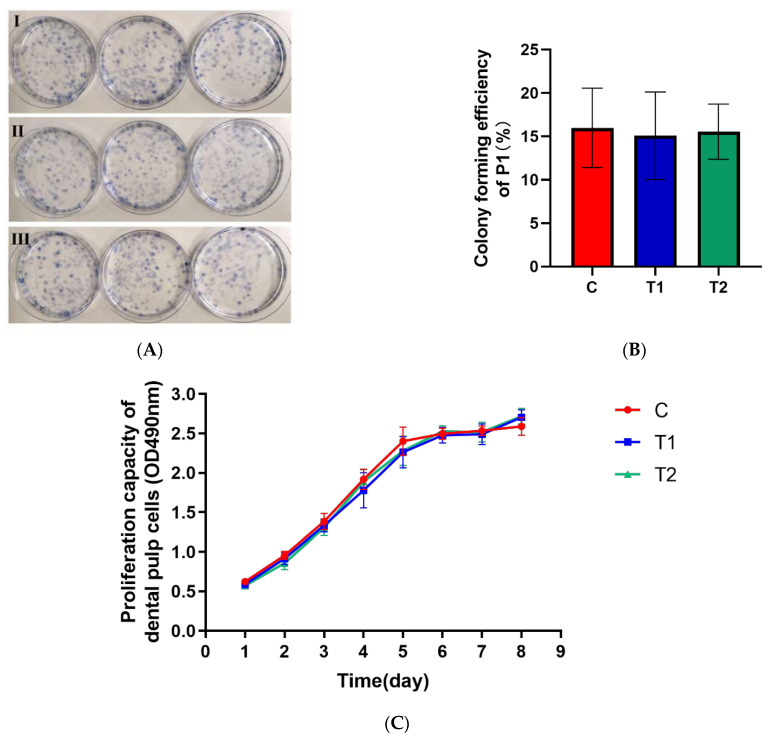
Proliferation ability of primary dental pulp cells in each group. (**A**) Giemsa staining results of dental pulp cells. Ⅰ: C group; Ⅱ: T1 group; Ⅲ: T2 group. (**B**) Colony-forming efficiency. (**C**) Cell growth curve (MTS assay).

There was no significant difference in the cell proliferation ability, including cell colony-forming efficiency as well as the growth curve among the three groups (*p* > 0.05) (Figure 5B,C).

#### 2.1.6. Effects of New Cryopreservation Strategy on the Differentiation Potential of Dental Pulp Cells

**Figure 6:**
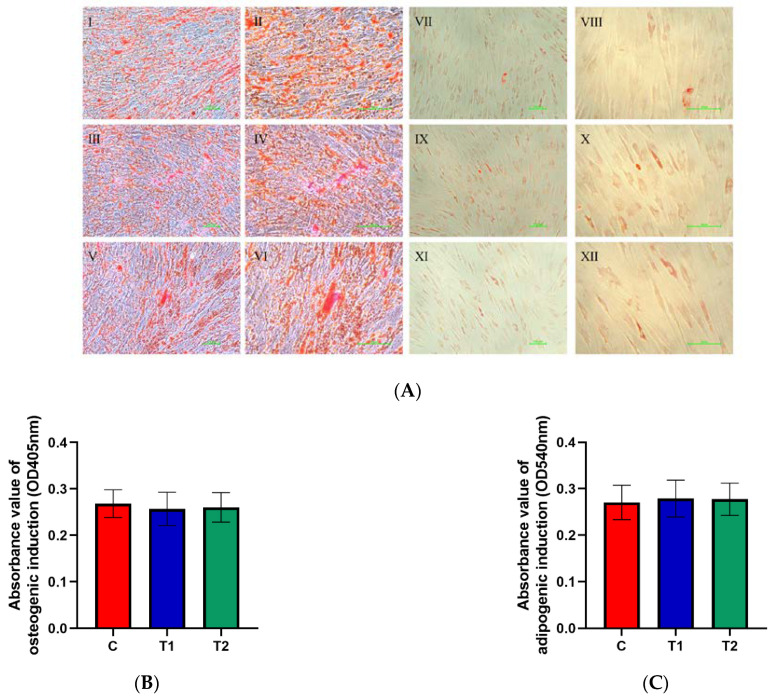
Differentiation potential of dental pulp cells. (**A**) Osteogenic and adipogenic differentiation results of dental pulp cells. Ⅰ–Ⅵ: Osteogenic differentiation results. Ⅰ: C group (×40); Ⅱ: C group (×100); Ⅲ: T1 group (×40); Ⅳ: T1 group (×100); Ⅴ: T2 group (×40); Ⅵ: T2 group (×100). Ⅶ–Ⅻ: Adipogenic differentiation results. Ⅶ: C group (×40); Ⅷ: C group (×100); Ⅸ: T1 group (×40); Ⅹ: T1 group (×100); Ⅺ: T2 group (×40); Ⅻ: T2 group (×100). (**B**) The absorbance value at 450 nm of dissolved solution after Alizarin Red S staining in the three groups. (**C**) The absorbance value at 540 nm of dissolved solution after Oil Red O staining in the three groups.

The cells from the third generation were stained after osteogenic and adipogenic induction for three weeks following the respective protocols (Figure 6A) and the corresponding absorbance was detected by a spectrophotometer at different wavelengths. The results illustrated that there was no difference among the three groups in terms of the ability of osteogenic and adipogenic differentiation (*p* > 0.05) (Figure 6B,C).

#### 2.1.7. Representative Gene Expression Profile of LPL, PPARG, ALP, RUNX2, COL I and OSC in Each Group

**Figure 7:**
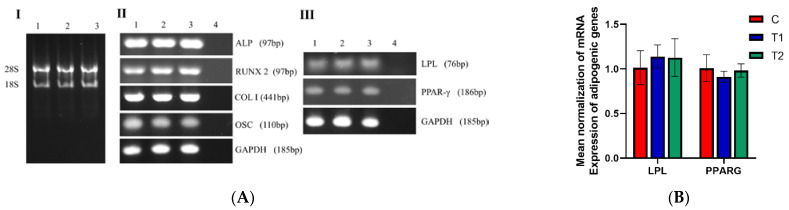

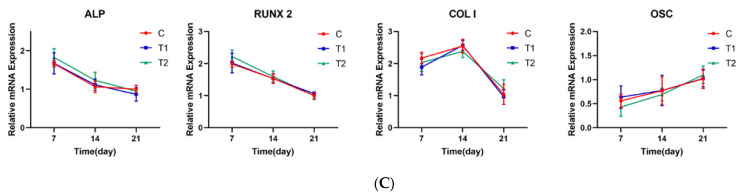
Representative gene expression profile in different groups. (**A**) Gel electrophoresis results. Ⅰ: RNA electrophoresis of three groups. 1: C group; 2: T1 group; 3: T2 group. Ⅱ: Electrophoresis results of osteogenic expression and reference genes in each group. 1: C group; 2: T1 group; 3: T2 group; 4: Negative control group. Ⅲ: Electrophoresis results of adipogenic expression and reference genes in each group. 1: C group; 2: T1 group; 3: T2 group; 4: Negative control group. (**B**) Quantitative real-time PCR results of adipogenic genes. (**C**) Quantitative real-time PCR results of osteogenic genes.

The relative expressions of two adipogenic and four osteogenic genes were not significantly different at the same time point among the three groups (*p* > 0.05). However, the relative expression of osteogenic genes changed with the time of osteogenic induction. (Figure 7A–C).

#### 2.1.8. ALP Assay Test to Identify the Osteogenic Activity of hDPSCs

**Figure 8:**
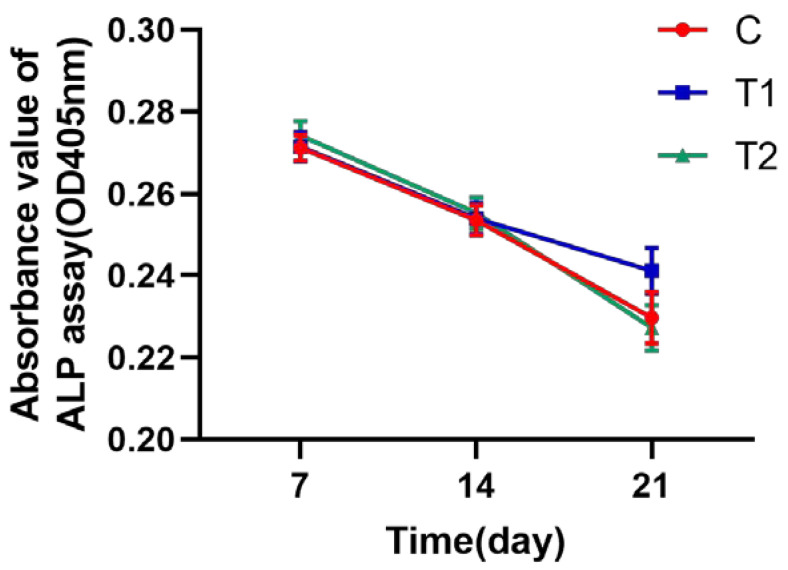
ALP assay results of dental pulp cells in each group.

There was no significant difference in the ALP activity among the three groups at the same time point. As the duration of osteogenic induction increased, ALP activity gradually decreased (*p* > 0.05, Figure 8).

## 3. Discussion

In this study, we devised a more accessible novel cryopreservation approach using varying concentrations of DMSO to preserve the third molars, which can be a source of hDPSCs for the donor. To evaluate the effect of the new cryopreservation strategy, 18 third molars were extracted and cell lineages were separated, then the difference between the frozen and unfrozen teeth was examined. It was demonstrated that the T1 and T2 DMSO frozen groups showed significantly longer times for the outgrowth of cells than the fresh dental pulp tissue, indicating that a higher dose of DMSO will extend the growth time of primary cells. Last but not least, the total number of primary cells yielded in the T1 and the T2 frozen groups was considerably lower than in the C group. After the first passage, however, the cryopreservation at −80 °C for one month showed no influence on the morphology of the dental pulp cells, including their cell size, cytoplasmic density and nucleus. The data of colony-forming efficiency, cell survival rate and the MTS test illustrated that the new cryopreservation method had no impact on dental pulp cell activity. In accordance with the International Cell Therapy Society’s (ISCT) guidelines, we identified the surface antigens of the hDPSCs cultured from the teeth that were frozen using novel cryopreservation techniques and discovered that CD45 and CD34 expression was negative in the three groups, while CD73, CD90 and CD105 expression was positive, which is similar to a previous study [5]. Since these cells’ surface antigens biologically match those of MSCs, cryopreservation techniques would not have a negative impact on the stemness of dental pulp cells. In addition, we noticed that the ability of dental pulp cells to differentiate into adipogenic and osteogenic tissues in cryopreservation groups was not significantly affected according to the detection of calcium and lipid deposits after 21 days of differentiation induction, and the relevant mRNAs—especially relevant osteogenic genes including ALP, RUNX2, COL I and OSC—were not observed to be downregulated at the same time point (day 7, day 14 and day 21). The ALP assay results among the three groups showed no significant difference, but the relative expression of osteogenic genes changed with the time of osteogenic induction, demonstrating that dental pulp cells in the DMSO frozen groups retained their differentiation capacity after the passage.

The present study by our group showed that the new cryopreservation strategy inevitably reduced the harvest of primary pulp cells and increased their initial appearance time, which may be due to the cryopreservation approach reducing the contact between the pulp tissue and the cryoprotectants that ensure the integrity of the teeth, resulting in an insufficient number of cells being protected. Furthermore, being one of the most widely used cryoprotectants, DMSO concentrations below 10% can be mildly hazardous, despite its ability to decelerate the ice-crystal-formation rate in cells and preserve the structural and functional integrity of cells after unfreezing [22,27]. The passaged pulp cells’ growth proliferation and differentiating capacities, on the other hand, were not considerably harmed by freezing. This might be due to a month-long freezing period not being too lengthy; therefore, cell damage was not obvious. In addition, the dental tissue of the donors selected by this experiment had a high cell volatility, allowing the cells to withstand physical and chemical stimulation.

There is a significant demand for bone-regeneration therapy in individuals over 40 years of age due to dental implant treatment, tumor surgery, trauma, and periodontitis-induced alveolar bone loss [28]. In this regard, hDPSCs are considered a promising source of cells for regenerative medicine and tissue engineering [29]. However, research has shown that the quantity of MSCs and their ability of self-renewal are reduced as age increases [30]. The proliferation and differentiation ability of MSCs also decreases with age due to telomere shortening [31], DNA damage and epigenetic changes in transcriptional regulation [32,33]. In addition, increased secondary dentinogenesis and root-canal mineralization in older individuals resulted in the significant shrinkage of pulp tissue, which further increased the difficulty of collecting hDPSCs [34]. Therefore, preserving patients’ hDPSCs in advance at a young age is one of the keys to ensuring their stem-cell function (including proliferation and differentiation ability). With the preservation method used in our experiment, we can cryopreserve and store as many hDPSCs with the best stemness and the highest proliferative capacity as possible.

A previous study by our lab has shown that dental pulp tissue can be cryopreserved in a 5% and a 10% DMSO culture media at −196 °C for one month, with no significant difference in the proliferation, cell growth, and differentiation capacity of hDPSCs between the frozen and unfrozen groups [35]. This conclusion serves as a useful guide for the large-scale and long-term preservation of dental pulp tissue. However, there is still a risk of tissue contamination due to the constrained settings, primarily the lack of a sterile atmosphere in the operation blocks of clinics and hospitals. Moreover, most dental offices lack liquid nitrogen cryopreservation chambers at −196 °C to preserve tissue and cells. Our new approach supports evidence from prior studies, which noted that hDPSCs stored at −80 °C in 10% DMSO for 1–5 years still retained very high capabilities [36,37]. This is also partly consistent with a study showing that hDPSCs can be preserved at −85 °C for six months without a loss of function [38]. Ginani F et al. also discovered that after six-month storage at −80 °C, cells from human exfoliated deciduous teeth could retain similar properties in terms of the cell viability and proliferation rate in 10% DMSO for up to six months [39], suggesting that the method of the cryopreservation of whole teeth may still be suitable for the preservation of deciduous teeth.

The ability of stem cells to multi-differentiate is a prerequisite for their therapeutic use. Because of their ability to develop into endoderm, mesoderm, and ectoderm lineages, dental mesenchymal stem cells are considered an ideal source of stem cells in cell engineering and tissue regeneration. Yanasse et al. mixed hDPSCs with platelet-rich plasma (PRP) to form a stem-cell scaffold and found a significant improvement in articular cartilage repair in a rabbit model [40]; Wang et al. injected the cultured secretome or vehicle (DMEM) of hDPSCs into a mouse model of amyotrophic lateral sclerosis (ALS) and found a significant increase in the number of days of survival after the onset of the disease and in the total life span of the mice [41]; Li et al. cultured and intravenously transplanted hepatocyte growth factor (HGF)-transformed hDPSCs into a rat model of ulcerative colitis (UC) and found that HGF–DPSCs could inhibit inflammatory responses by transdifferentiating into intestinal stem cell (ISC)-like cells, promoting ISC-like cell proliferation, inhibiting inflammatory responses and reducing oxidative stress injury [42]; Hata et al. injected hDPSCs into diabetic polyneuropathic nude mice after culture and found that they significantly improved delayed nerve-conduction velocity, reduced blood flow and increased the sensory perception threshold [43]. Apart from that, hDPSCs have a significant advantage over other stem-cell sources in terms of accessibility. Third molars are the most common source of teeth for use in stem-cell cryopreservation compared to other teeth. It was reported by Carter et al. that the prevalence of wisdom teeth ranges from 18–68%, with 41% of those being diagnosed as mesioangular impaction [44], which were frequently suggested for prophylactic extraction by dentists. In this study, the teeth selected were incompletely erupted, impacted third molars, which do not affect the oral chewing function when extracted. Impacted third molars can cause swelling and ulceration of the surrounding gingival area, root damage to the second molars, decay of the second molars, and gingival and skeletal disease around the second molars. Impacted third molars are also related to pathological changes such as pericoronitis, root resorption, periodontal disease, caries, and the development of cysts or tumors [45]. In the long term, the retention of impacted wisdom teeth may enhance the risk of pathology in the surrounding structures, and their removal at later ages may result in more frequent and serious complications [46]. Therefore, the preventive extraction of asymptomatic healthy wisdom teeth, whether impacted or fully erupted, has long been regarded as appropriate care [47,48]. In addition to impacted third molars, the second common source of hDPSCs is premolars [23], especially those extracted during orthodontic treatment for severe crowding and Class II malocclusions. The age of these patients is usually around 10 to 16 years [49]. Multiple teeth are the third most common source of hDPSCs [23]. Therefore, from a biological point of view, any other healthy tooth, including the above three categories, may be used as a source of hDPSCs. Additionally, the premolars extracted during orthodontic treatment are another frequent source of dental pulp cells [23]. Rather than being regarded as biological waste, viable dental pulp cells could be a dependable and universal source of stem cells that can be broadly applied to organ reconstruction and tissue regeneration if the teeth can be preserved appropriately following extraction surgeries. Amongst the conventional cryopreservation processes, the most critical step can be the extraction of the dental pulp, in which an improper technique can easily lead to dental tissue contamination. Moreover, as storage time rises, the amount of pulp stem cells that can be separated from removed teeth decreases [50]. Studies have shown that dental pulp stem cells can remain active in vitro for up to one night or 12 h [51]. To put it another way, in a sterile operating environment, this is the shortest possible exposure time in vitro. Our study has proved that whole-tooth cryopreservation at −80 °C is achievable, which would boost the success rate of obtaining healthy dental pulp stem cells while minimizing the risk of contamination during operation after tooth extraction and maintaining the phenotype of primary cells. Furthermore, through this method, we were able to lower the requirements as well as the expense of hDPSC preservation, which creates better conditions for the establishment of dental stem-cell banks and future clinical applications.

However, due to the limited time and samples, additional research into the longest period of cryopreservation of complete teeth is problematic. If we figure out this problem, then the whole-tooth cryopreservation schedule would be more flexible, ensuring that they are therapeutically deployed at the optimal period for cell function. Our future research may be conducted on this topic to provide a deeper understanding of the preservation of hDPSCs.

## 4. Materials and Methods

### 4.1. Collection of Samples

**Figure 9:**
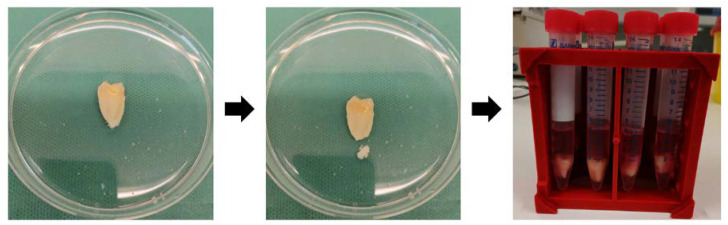
The procedure of teeth treatment in T1 and T2 frozen groups: 1/3 of the apical was removed and stored in the freezing medium containing DMSO.

**Figure 10:**
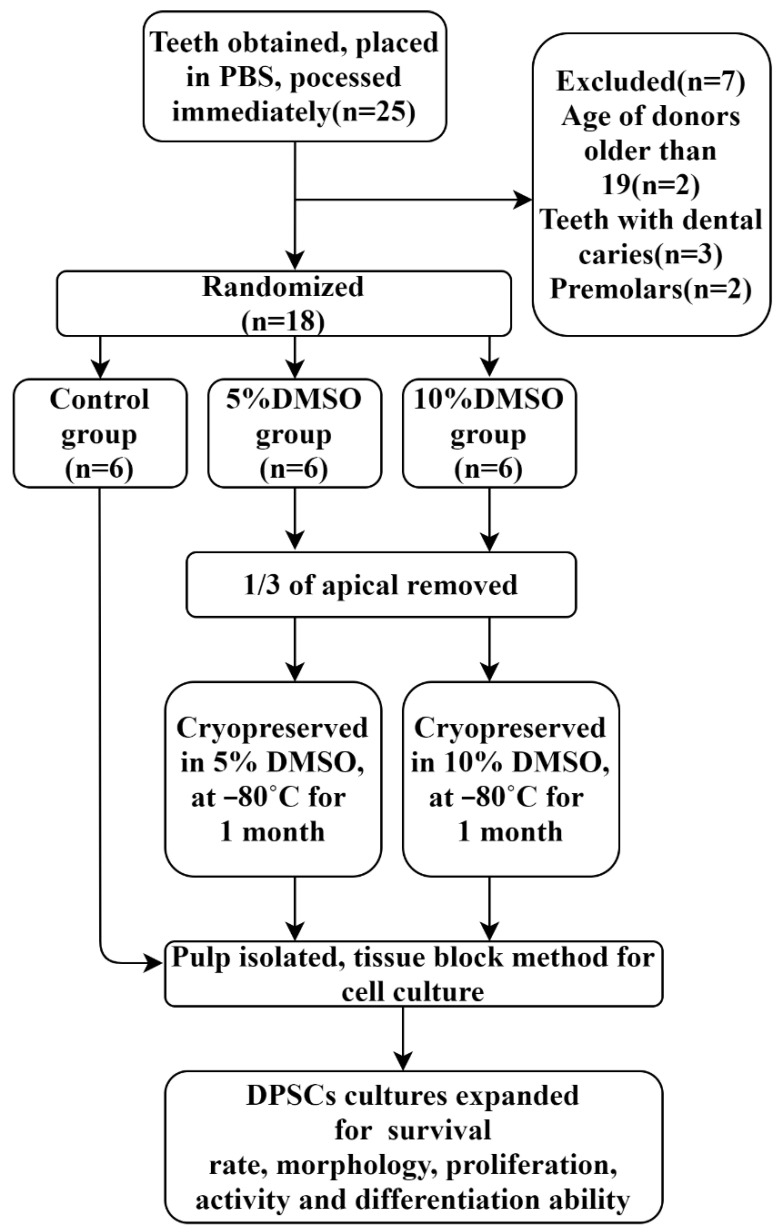
Flow diagram for teeth processing. DPSCs in six teeth were immediately cultured for cellular characterization experiments. After a month of cryopreservation, others were cultivated and examined.

Teeth extraction and cryopreservation: Between November 2019 and June 2020, 18 impacted third molars of healthy teenagers aged 15–19 years old were gathered in the Department of Oral and Maxillofacial Surgery of University Medical Center Hamburg-Eppendorf. The experimental protocol was authorized by the institutional review board of the medical chamber of Hamburg (IRB-vote # REC 1712/5/2008). The patients and their guardians completed the informed permission forms prior to surgeries and were taught to gargle with 1% H_2_O_2_ solution for 3 min. Furthermore, the standard sterilization procedures were followed during the whole process of operations. The extracted third molars were then removed and immediately placed in DMEM (Cat. NO. 41965-049, Gibco, Loughborough, UK) with 10% FBS (Cat. NO. 10500-064, Gibco, Paisley, UK) and penicillin (100 U/mL)/streptomycin (Cat. NO. 15140-148, Gibco, Paisley, UK) at 4 °C. Teeth were then soaked in 4 °C DPBS (Cat. NO. 14190-094, Gibco, Paisley, UK) solution (containing 3 × 10^5^ U/mL penicillin/streptomycin) for 30–60 min followed by experiments within 2–4 h (Figure 9). The whole progress of this experiments is depicted in a flow diagram (Figure 10).

### 4.2. Cryopreservation

The third molars were then allocated into 3 groups at random: a control group (C), a 5% DMSO group (T1), and a 10% DMSO group (T2). For the T1 and T2 groups, the corresponding concentration of DMSO (Cat. NO. 2308.0100, Geyer GmbH, Germany) medium was used to immerse the teeth. Previously, sterilized high-speed turbine drills were used to remove a section of the root from 1/3 of the apical in order for the DMSO cell-cryopreservation solution to have greater contact with the pulp tissue (Figure 8). After being stored in the refrigerator at 4 °C for 30 min and −20 °C for 1 h, the teeth in the T1 and T2 groups were then preserved in the −80 °C refrigerator for 1 month. The teeth in the C group, on the other hand, were split open using a sterilized high-speed turbine drill to expose the pulp. Thereafter, the intra-dental pulp tissues were cut into 0.5 mm^3^ tissue blocks and placed in cell culture plates.

### 4.3. Culture of hDPSCs

Tissues of each group were dispersed in 24-well plates. Each tissue block was positioned in its own well and dipped in 600 uL DMEM culture medium (with 10% FBS and 100 U/mL penicillin/streptomycin) after it affixed to the bottom. The plates with tissue blocks were then incubated at 37 °C in 5% CO_2_ incubators. Every day, an inverted microscope was used to evaluate cell adhesion and cultivation conditions. When confluency of colonies reached the optimal level, the cells were isolated with 0.05% Trypsin (Cat. NO. 25300-054, Gibco, Paisley, UK) and counted with cell-counting boards. The cells were passaged when a density of roughly 8 × 10^3^/cm^2^ was reached.

### 4.4. Assessment of Primary Cellular Morphology and Recording of Primary Cell Growth Time

Two trained and calibrated experimenters examined the pulp tissue daily with an inverted microscope, assessed the morphology of the primary cells and recorded the time from tissue block implantation to cell adhesion and expansion for each group. When the cells reached 80–90% confluency, the primary cells were digested and passaged.

### 4.5. Cell Yield Computation

After the primary cells reached 80–90% confluency, they were digested using 0.05% trypsin for 3 min. The trypsinization process was halted with an equivalent volume of DMEM culture medium as soon as the cellular morphology was observed to be spherical and floating. Afterward, the hDPSCs were transferred into 50 mL tubes and centrifuged at 1000 rpm for 10 min at room temperature. The supernatant was discarded and the cells were suspended again in DMEM culture medium. The number of cells was calculated with cell-counting plates. Primary cell quantity = Total cell number/4 × dilution factor × 104 × volume of cell suspension

### 4.6. Flow Cytometry

Dental pulp cells were collected for flow cytometry. Specific PE-conjugated antibodies including CD34 (Cat. NO. 343505, Biolegend, San Diego, CA, USA), CD45 (Cat. NO. 304007, Biolegend, San Diego, CA, USA), CD73 (Cat. NO. 344003, Biolegend, San Diego, CA, USA), CD90 (Cat. NO. 328109, Biolegend, San Diego, CA, USA) and CD105 (Cat. NO. 323205, Biolegend, San Diego, CA, USA) were chosen to test the cells following the protocol from the manufacturer. Each tube containing 2 × 10^5^ cells received 100 μL of dye liquid containing 1:1000 live/dead dye and 1:100 antibody, which was then incubated for 20 min at room temperature. After being washed with PBS, cells were detected with BD LSRFortessa cell analyzer (Becton Dickinson Bioscience, Becton, USA) and BD FACSDiva software V6.1.3 (Becton Dickinson Bioscience, USA). Data were further analyzed with FlowJo software V10.0.7 (Treestar Inc., Ashland, OR, USA).

### 4.7. Colony-Forming Efficiency

A total of 1 × 10^3^ cells of each group from the first generation were seeded on 10 cm-diameter culture dishes. The culture medium was changed every 3 days. The medium was removed two weeks later, and the cellular colonies were fixed in pure methanol for 10–15 min, rinsed three times with DPBS, and stained with Giemsa Solution (Cat. NO. 48900, Sigma-Aldrich, Schaffhausen, Switzerland) for 10 min. The colonies of dental pulp cells with a number greater than 50 were recorded as being available. Three parallel dishes were set for 1 sample. Colony-forming efficiency = number of available colonies/number of seeded cells × 100%

### 4.8. Cell Survival Rate

Trypan Blue staining: Cells from the first generation in the three groups were stained with 0.4% Trypan Blue (Cat. NO. 15250-061, Gibco, Paisley, UK) for 2 min and then observed using the inverted microscope. Within 500 dental pulp cells, the number of unstained living cells was recorded and the cell survival rates were computed. Cell survival rate of Trypan Blue = number of unstained living cells/500 × 100%

Live–dead staining: Cells from the first generation of each group were seeded on TCC (tissue culture coverslips, Cat. NO. 83.1840.002, Sarstedt, Nümbrecht, Germany) at a density of 8 × 10^4^/mL in 12-well plates, corresponding to 8 × 10^4^ cells per well, and were incubated at 37 °C in 5% CO_2_ incubators for 4 h. Next, 60 μL of propidium iodide (PI) (50 μg/mL in PBS) and 500 μL fluorescein diacetate (FDA) working solution (20 μg/mL) were added to each well. After 3 min of incubation at room temperature and rinsing with DPBS, samples were observed with a fluorescence microscope (Nikon ECLIPSE Ti-S/L100, Düsseldorf, Germany). The number of live cells, which were stained in green, were counted. The cell survival rate was calculated. Cell survival rate of live–dead staining = number of green-stained cells/number of total cells × 100%

### 4.9. Proliferation Testing with MTS Assay

Cells from the third generation were seeded in 96-well plates at a density of 2 × 10^4^/mL, equating to 2 × 10^3^ cells per well, and were incubated at 37 °C in 5% CO2 incubators. The proliferation of cells was measured with the MTS assay (Cat. NO. G1111, Promega, Madison, USA) for 8 continuous days in total. Cells from 3 wells of each sample in every group were subjected to MTS colorimetric analysis. Next, 20 μL of MTS reagent was added to each well and the absorbance was detected after 3 h of incubation using a microplate reader (Thermo Fisher Scientific, Waltham, MA, USA) at the wavelength of 490 nm.

### 4.10. Differentiation Potential Assessment

Adipogenic differentiation: Cells from the third generation were cultured at a density of 2×10^4^/mL, corresponding to 4 × 10^4^ per well in 6-well plates. EVE Automatic Cell Counter (NanoEntek, Seoul, Korea) was used to calculate the concentration of the cell suspension in each group 3 times. Then, the cell suspension was plated at the same volume into 6-well plates after normalizing the concentration of cells in each group to ensure that the number of cells added to each well and the volume of culture medium were the same. The cells were grown in adipogenesis-induction medium (DMEM containing 10% FCS, 5 μg/mL insulin, 0.5 mmol/L 3-isobutyl-1-methylxanthine, and 10 μmol/L dexamethasone) for 3 weeks after reaching 60–70 percent confluency, and the medium in the wells was replaced every 3 days. On the 22nd day, the cells were fixed with paraformaldehyde (PFA; Electron Microscopy Sciences, Fort Washington, PA, USA) for 20 min and rinsed 3 times with DPBS. The stimulated cells were then stained with 0.5% Oil Red O solution (Cat. NO. O1391-250ML, Sigma-Aldrich, St. Louis, MS, USA) for 10–15 min. After staining, cells were rinsed 3 times with DPBS and the stained lipid droplets in the cytoplasm were seen and photographed using an inverted microscope.

Adipogenic differentiation quantitative analysis: To eliminate the remaining staining solution, stained cells in plates were washed three times with DPBS. Thereafter, 1 mL isopropanol (Cat. NO. 34965-1L, Honeywell, North Carolina, Charlotte, US) was added to each well to dissolve the lipid droplets, and the plates were gently shaken until the solution was equally colored. Then, the solution was transferred into 96-well plates at 100 μL well and detected using a microplate reader (Thermo Fisher Scientific, Waltham, MA, USA) at a wavelength of 540 nm. Three parallel groups were set for 1 sample.

Osteogenic differentiation: Cells from the third generation were cultured at a density of 2 × 10^4^/mL, corresponding to 4 × 10^4^ per well in 6-well plates. EVE Automatic Cell Counterwas used to calculate the concentration of cell suspension in each group 3 times. Then, the cell suspension was plated at the same volume into 6-well plates after normalizing the concentration of cells in each group to ensure that the number of cells added in each well and the volume of culture medium were the same. After attaining 60–70 percent confluency, the cells were cultured for 3 weeks in osteogenic-induction medium (DMEM with 10% FCS, 10 mmol/L glycerophosphate, 5 mmol/mL ascorbic acid, and 1 mol/L dexamethasone). The medium was changed every 3 days. On the 22nd day, the cells were fixed with paraformaldehyde for 20 min and washed 3 times with DPBS. The stimulated cells were then stained for 10–15 min with 0.1% Alizarin red S (Cat. NO. GT6383, Glentham, Germany) solution. After staining, cells were rinsed 3 times with DPBS and the inverted microscope was used to observe and take pictures of the stained calcium nodules.

Osteogenic differentiation quantitative analysis: To remove leftover staining solution, stained cells in plates were washed three times with DPBS. Following that, 750 μL of 10% acetic acid (Cat. NO. 2289.1000, Geyer GmbH, Stuttgart, Germany) was transferred into each well and the plates were gently shaken to completely dissolve the stained calcium nodules. To neutralize the acetic acid, an equivalent amount (750 μL) of 10% ammonium hydroxide was applied. The solution was transferred into 96-well plates at 100 μL/well and detected using a microplate reader at a wavelength of 405 nm. Three parallel groups were set for 1 sample.

### 4.11. Osteogenic Activity with ALP Assay

Following the ALP assay kit instructions (ab83369 Alkaline Phosphatase Assay Kit, Cambridge, UK), 80 µL of the supernatant of each group after osteogenic induction was added to 96-well plates for the ALP assay. Then, 20 µL of stop solution was added to the sample background control wells to terminate ALP activity in these samples. The samples were mixed well by pipetting up and down. Next, 50 µL of 5 mM pNPP solution was added to each well containing the sample and background sample controls. Then, 10 µL of ALP enzyme solution was added to each pNPP standard well. Plates were incubated at 25 °C for 60 min while protected from light. The reaction was ceased in sample wells and standard wells by adding 20 µL of stop solution. The output at OD 405 nm was measured on a microplate reader. The ALP activities of each group were detected at day 7, day 14 and day 21, respectively.

### 4.12. Gene Expression Detection

TRIzol reagent (Cat. NO. 15596026, Ambion, Austin, TX, USA) was used to extract total RNA from differentiated dental pulp cells, which was subsequently quantified using a spectrophotometer (Cat. NO. 51119700DP, Thermo Fisher, Singapore) and 1 percent agarose gel electrophoresis. The isolated total RNA was reverse transcribed to cDNA for the reverse-transcription PCR as well as real-time quantitative PCR analysis using GoScriptTM RT reagent Kit (Cat. NO. A5001, Promega, Madison, WI, USA) and Luna^®^ Universal One-Step RT-qPCR Kit (Cat. NO. E3005, New England biolabs INC, Ipswich, MA, USA) according to the manufacturer’s protocol. Primers were designed on Primerbank. Lipoprotein lipase (LPL) and peroxisome proliferator-activated receptor-γ (PPAR-γ) were selected as adipogenic-specific genes; otherwise, alkaline phosphatase (ALP), runt-related transcription factor 2 (RUNX2), type I collagen (COL I) and osteocalcin (OSC) were selected as osteogenic genes and their expressions were detected at day 7, day 14 and day 21. GAPDH was selected as the reference housekeeping gene of each sample. The relative quantity of mRNAs was calculated with the 2^−ΔΔCt^ method after normalization. Table 1 shows the primer sequences as well as the length of the products.

### 4.13. Statistical Analysis

Variance in the mean values across the three groups was analyzed using a student *t*-test. Statistical significance was defined as a *p*-value of less than 0.05. For statistical analysis, SPSS 25.0 software (SPSS Inc., Chicago, IL, USA) and Graphpad Prism V9.0 (GraphPad Software, San Diego, CA, USA) was utilized.

## 5. Conclusions

Our research aimed to examine the effect of cryopreserving whole teeth with 5% and 10% DMSO on hDPSCs. The results of our study indicated that the new strategy of sample freezing and unfreezing had a negative effect on the initial culture stage of dental pulp cells, which prolonged the growth and culture time of dental pulp cells. However, the results of the cell experiments on the third generation showed that the biological activity of the experimental dental pulp stem cells was almost not affected. The hDPSCs maintained a high proliferation and differentiation ability. The new strategy, on the other hand, effectively reduced the exposure time of the samples to aseptic conditions, which can reduce the chance of infection. More importantly, the new cryopreserved method effectively lowers the tissue-preservation requirements, so that the common dental practice can preserve the samples in time, too. This maximizes the freshness of the pulp tissue and the biological activities of the dental pulp stem cells. Although the small sample size did not allow us to further investigate the maximum duration of the cryopreservation of whole teeth, our study shows the feasibility of the new cryopreservation method, which can provide a theoretical basis for the establishment of a dental stem-cell bank and create better conditions for future stem-cell therapy. 

## Figures and Tables

**Table 1:** Primer sequences of adipogenic- and osteogenic-induced gene expression.

Primer	Direction	Sequence	Length of Products (bp)
LPL	Forward	ACAAGAGAGAACCAGACTCCAA	76
	Reverse	GCGGACACTGGGTAATGCT	
PPAR-γ	Forward	GGGATCAGCTCCGTGGATCT	186
	Reverse	TGCACTTTGGTACTCTTGAAGTT	
ALP	Forward	ACTGGTACTCAGACAACGAGAT	97
	Reverse	ACGTCAATGTCCCTGATGTTATG	
RUNX 2	Forward	TGGTTACTGTCATGGCGGGTA	97
	Reverse	TCTCAGATCGTTGAACCTTGCTA	
Type I collagen	Forward	GGACACAATGGATTGCAAGG	441
	Reverse	AACCACTGCTCCACTCTGG	
Osteocalcin	Forward	GGCGCTACCTGTATCAATGG	110
	Reverse	GTGGTCAGCCAACTCGTCA	
GAPDH	Forward	GAGTCAACGGATTTGGTCGT	185
	Reverse	GACAAGCTTCCCGTTCTCAG	

## Data Availability

The data presented in this study are available on request from the corresponding author.
